# ^64^Cu-ATSM internal radiotherapy to treat tumors with bevacizumab-induced vascular decrease and hypoxia in human colon carcinoma xenografts

**DOI:** 10.18632/oncotarget.21323

**Published:** 2017-09-28

**Authors:** Yukie Yoshii, Mitsuyoshi Yoshimoto, Hiroki Matsumoto, Takako Furukawa, Ming-Rong Zhang, Masayuki Inubushi, Atsushi B. Tsuji, Yasuhisa Fujibayashi, Tatsuya Higashi, Tsuneo Saga

**Affiliations:** ^1^ National Institute of Radiological Sciences, National Institutes for Quantum and Radiological Science and Technology, Chiba, Japan; ^2^ Division of Functional Imaging, National Cancer Center Hospital East, Kashiwa, Japan; ^3^ Research Centre, Nihon Medi-Physics Co., Ltd., Sodegaura, Japan; ^4^ Department of Radiological and Medical Laboratory Sciences, Nagoya University Graduate School of Medicine, Nagoya, Japan; ^5^ Department of Nuclear Medicine, Kawasaki Medical School, Kurashiki, Japan; ^6^ Department of Diagnostic Radiology, Kyoto University Hospital, Kyoto, Japan

**Keywords:** ^64^Cu-ATSM, angiogenesis, bevacizumab, hypoxia, vascular decrease

## Abstract

Bevacizumab, an anti-vascular endothelial growth factor (VEGF) antibody, is an antiangiogenic agent clinically used for various cancers. However, repeated use of this agent leads to tumor-decreased vascularity and hypoxia with activation of an HIF-1 signaling pathway, which results in drug delivery deficiency and induction of malignant behaviors in tumors. Here, we developed a novel strategy to treat tumors with bevacizumab-induced vascular decrease and hypoxia using ^64^Cu-diacetyl-bis (*N*^4^-methylthiosemicarbazone) (^64^Cu-ATSM), a potential theranostic agent, which possesses high tissue permeability and can target over-reduced conditions under hypoxia in tumors, with a human colon carcinoma HT-29 tumor-bearing mouse model. The long-term treatment with bevacizumab caused decreased blood vessel density and activation of an HIF-1 signaling pathway; increased uptake of ^64^Cu-ATSM was also observed despite limited blood vessel density in HT-29 tumors. *In vivo* high-resolution SPECT/PET/CT imaging confirmed reduced vascularity and increased proportion of ^64^Cu-ATSM uptake areas within the bevacizumab-treated tumors. ^64^Cu-ATSM therapy was effective to inhibit tumor growth and prolong survival of the bevacizumab-treated tumor-bearing mice without major adverse effects. In conclusion, ^64^Cu-ATSM therapy effectively enhanced anti-tumor effects in tumors with bevacizumab-induced vascular decrease and hypoxia. ^64^Cu-ATSM therapy could represent a novel approach as an add-on to antiangiogenic therapy.

## INTRODUCTION

Angiogenesis is essential for tumor growth and is a useful target for cancer treatment [1]. Vascular endothelial growth factor (VEGF) is a key component of angiogenesis and the anti-VEGF antibody bevacizumab is widely used in clinical practice as an antiangiogenic agent for many types of cancers such as colorectal, lung, and breast cancer, as well as glioblastoma [1]. Bevacizumab treatment combined with standard chemotherapy is known to prolong survival in patients by inhibiting tumor growth [2]. Clinical studies indicated that the efficacy of bevacizumab therapy is not long-lasting, although bevacizumab treatment initially shows positive effects [3]. Previous studies reported that bevacizumab treatment decreases blood vessel density and induces low perfusion in tumor tissues, which leads to ineffectiveness of bevacizumab treatment via reducing drug delivery [4, 5]. In addition to this, continued use of antiangiogenic agents generates intratumoral hypoxia with stimulation of hypoxia-inducible factor-1 (HIF-1) signaling pathway, which leads to tumor regrowth and metastasis [6]. In order to improve the efficacy of bevacizumab treatment, additional strategies are required to treat tumors with bevacizumab-induced vascular decrease and hypoxia.

In this study, we focused on ^64^Cu-diacetyl-bis (*N*^4^-methylthiosemicarbazone) (^64^Cu-ATSM), a promising theranostic agent with high tissue permeability and targeting of over-reduced state under hypoxia within tumors [7–17]. Cu-ATSM labeled with various Cu radioisotopes, such as ^60^Cu, ^62^Cu, and ^64^Cu, has been originally developed as an imaging agent targeting the hypoxic regions in tumors for use with positron emission tomography (PET) [7, 9, 13–15, 17, 18]. Preclinical studies have revealed that Cu-ATSM rapidly diffuses into cells and tissues even in low perfusion areas and is trapped within cells under highly reduced conditions such as hypoxia and that tumor uptake of Cu-ATSM shows good correlation with the HIF-1α expression [9, 14, 15, 17, 19–21]. In recent years, clinical PET studies using radiolabeled Cu-ATSM have been conducted for many types of cancers throughout the world. These clinical studies have shown that Cu-ATSM uptake is associated with high HIF-1α expression, therapeutic resistance, metastatic potential, and poor prognosis in tumors [7, 22–25]. Thus, the previous studies indicate a connection between Cu-ATSM uptake and hypoxia with activation of an HIF-1 signaling pathway.

^64^Cu-ATSM can be used not only as a PET imaging agent but also as a radiotherapeutics against tumors, since ^64^Cu shows β^+^ decay (0.653 MeV, 17.4%) as well as β^-^ decay (0.574 MeV, 40%) and electron capture (42.6%). The photons from electron-positron annihilation can be detected by PET, while the β^-^ particles and Auger electrons emitted from this nuclide can damage tumor cells [13, 16, 18, 26]. ^64^Cu-ATSM reduces the clonogenic survival of tumor cells under hypoxia by inducing post-mitotic apoptosis [13]. This is caused by heavy damage to DNA via high-linear energy transfer (LET) Auger electrons emitted from ^64^Cu [27]. An *in vivo* study using tumor-bearing hamsters demonstrated that ^64^Cu-ATSM treatment increased survival time [18]. These previous studies support the feasibility of ^64^Cu-ATSM treatment of low vascular and hypoxic tumors, with the high permeability and high-LET radiation.

Here, we hypothesized that ^64^Cu-ATSM would be effective against tumors with bevacizumab-induced vascular decrease and hypoxia, and examined the efficacy of ^64^Cu-ATSM using a mouse model.

## RESULTS

### Tumor blood vessel density, activation of an HIF-1 signaling pathway, and ^64^Cu-ATSM uptake in bevacizumab-treated HT-29 tumors

**Figure 1:**
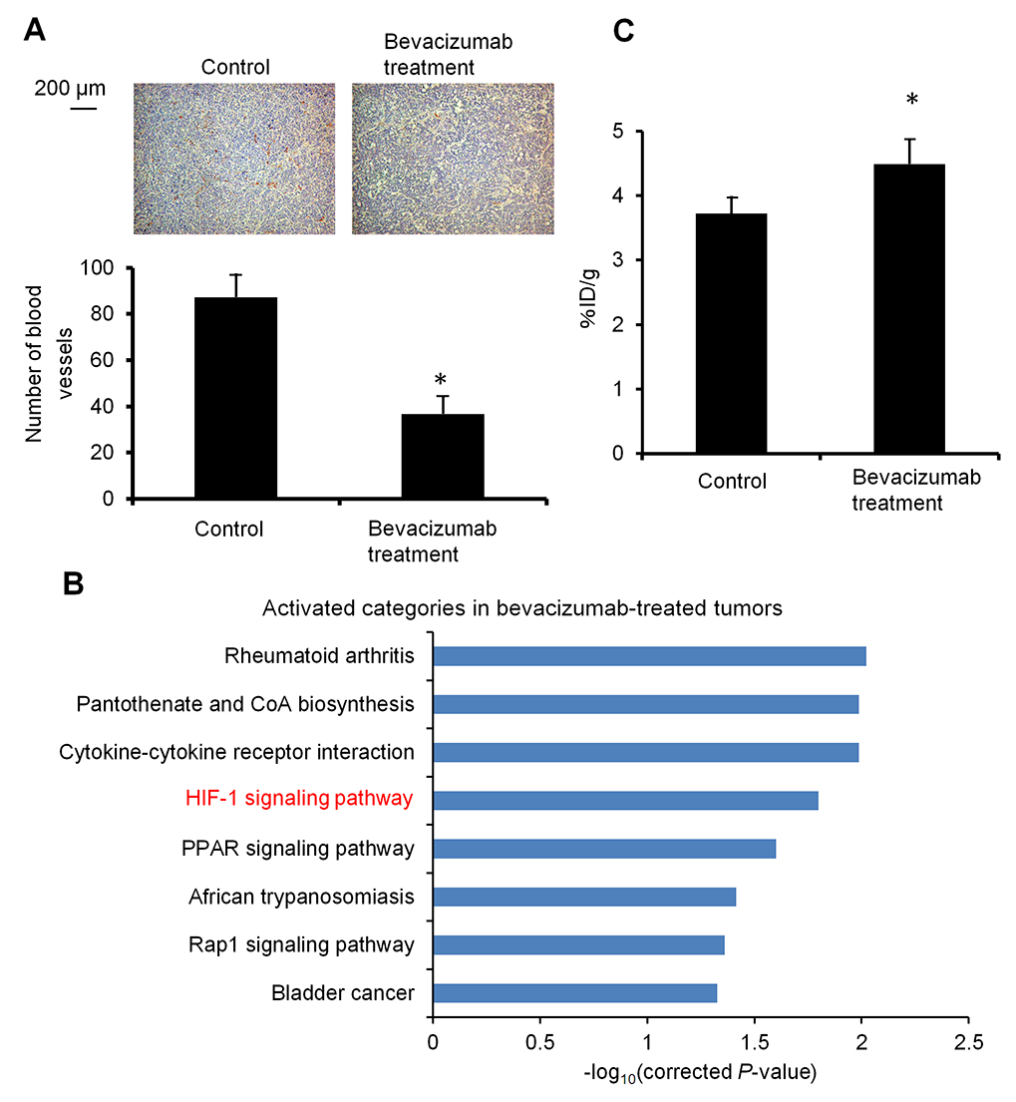
Characterization of bevacizumab-treated HT-29 tumors **(A)** Tumor blood vessel density. Representative CD31 immunohistochemical staining images of bevacizumab-treated HT-29 tumors (right) and untreated control (left) are shown (upper). Cells expressing CD31 were stained brown. Number of blood vessels in the observation areas in each tumor (n = 4) (lower). Values are shown as mean ± SD. *P < 0.05. **(B)** Activation of an HIF-1 signaling pathway. Activated pathways in the bevacizumab-treated HT-29 tumors, compared to the untreated control, are shown (n = 3). The pathways significantly activated (*P* < 0.05) were obtained by pathway analysis. HIF-1 signaling pathway, related to hypoxia, was activated in the bevacizumab-treated tumors. **(C)** Tumor uptake of ^64^Cu-ATSM. Tumor ^64^Cu-ATSM uptake in the bevacizumab-treated HT-29 tumors and control is shown (n = 4). Values are indicated as mean ± SD. *P < 0.05.

To make a bevacizumab-treated tumor model with vascular decrease and hypoxia, HT-29 tumor-bearing mice were treated with bevacizumab (5 mg/kg twice a week) for 3 weeks. The dose and duration of bevacizumab administration was decided based on a previous report, which studied tumor growth with HT-29 xenograft mice under bevacizumab treatment [5]. Prior to the treatment study, we examined the characteristics of this model. Blood vessel density was examined with CD31 immunohistochemistry in the bevacizumab-treated HT-29 tumors and bevacizumab-untreated control. Figure 1A shows representative images of CD31 staining in each tumor and the blood vessel density analyzed based on these. The bevacizumab-treated tumors showed significantly reduced blood vessel density, compared to the control (0.42-fold) (*P* < 0.05). To examine the hypoxia-induced reaction in the bevacizumab-treated tumors, we evaluated activation of the HIF-1 signaling pathway by DNA microarray-based analysis. Figure 1B shows the pathways significantly activated in the bevacizumab-treated tumors compared to the control. We found that an HIF-1 signaling pathway, which responds to hypoxia [28], was activated in the bevacizumab-treated tumors. Tumor uptake of ^64^Cu-ATSM was compared in mice with the bevacizumab-treated HT-29 tumors and the control mice. Tumor ^64^Cu-ATSM uptake was 1.2-fold higher in bevacizumab-treated tumors [the percentage injected dose per gram of tissue (%ID/g) =4.5 ± 0.5] compared to the control (%ID/g=3.7 ± 0.3) (*P* < 0.05) (Figure 1C). This demonstrated that the bevacizumab-treated tumors accumulated ^64^Cu-ATSM despite decreased blood vessel density.

### *In vivo* imaging with simultaneous single-photon emission computed tomography/positron emission tomography/computed tomography (SPECT/PET/CT) for intratumoral vascularity and ^64^Cu-ATSM uptake regions

**Figure 2:**
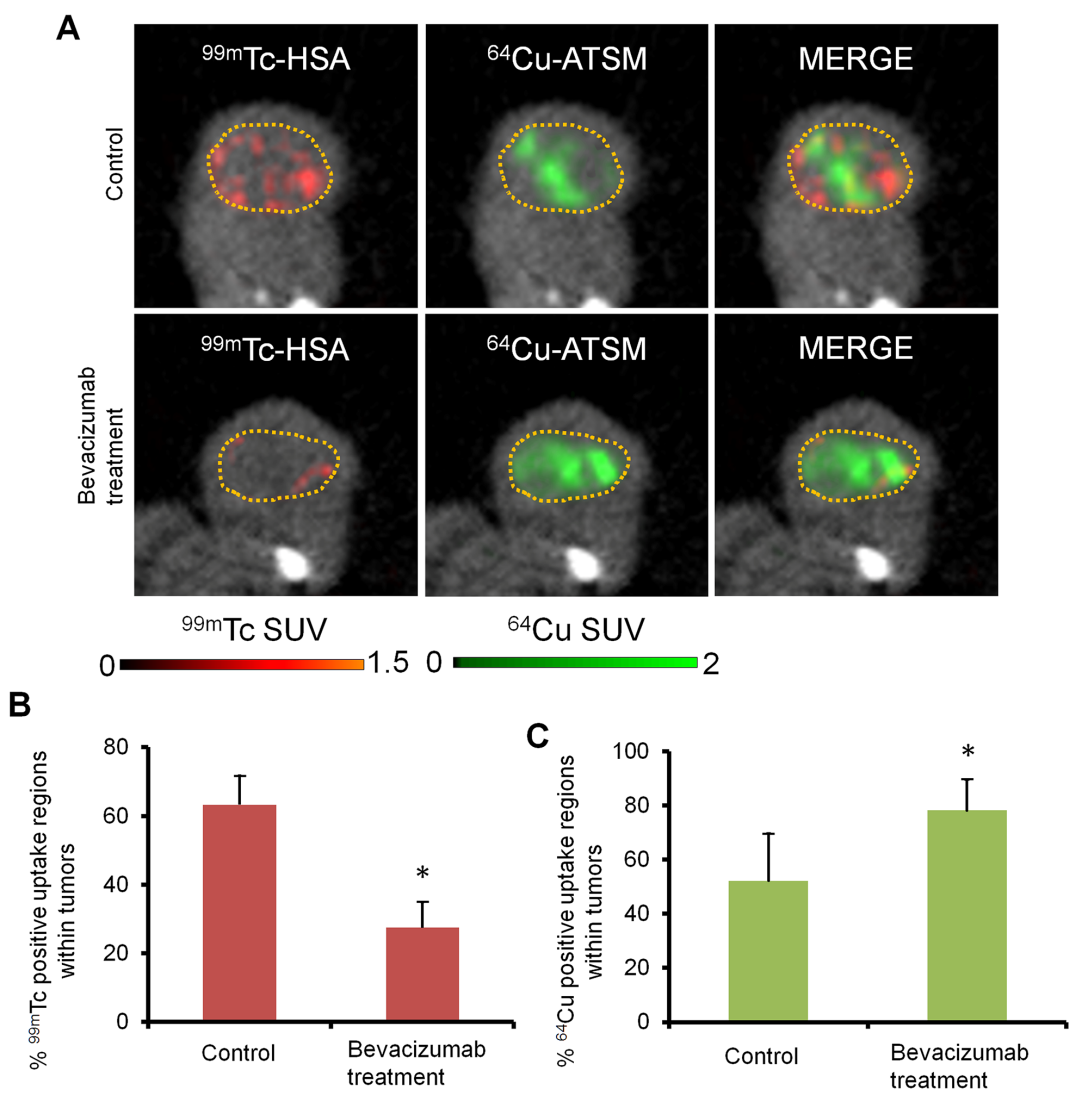
Simultaneous SPECT/PET/CT imaging for intratumoral vascularity and hypoxia **(A)** Representative images of the bevacizumab-treated tumor and untreated control, detected by dual-isotope SPECT/PET/CT with a blood pool-detecting agent^99m^Tc-HSA and a hypoxia-detecting agent ^64^Cu-ATSM. Dotted yellow circles, tumor regions; red, ^99m^Tc-HSA; green, ^64^Cu-ATSM. **(B)** and **(C)** % ^99m^Tc and ^64^Cu positive uptake regions (left and right, respectively) within tumors obtained from the analysis of SPECT/PET/CT images (n = 4). Values are shown as mean ± SD. *P < 0.05.

To further investigate intratumoral vascularity and ^64^Cu-ATSM uptake regions, *in vivo* high-resolution dual-isotope simultaneous SPECT/PET/CT imaging [29] with a blood pool-detecting SPECT agent,^99m^Tc-labeled human serum albumin (^99m^Tc-HSA) and a hypoxia-detecting PET agent,^64^Cu-ATSM, was conducted with bevacizumab-treated HT-29 tumors and the bevacizumab-untreated control. Figure 2A shows representative images and Figure 2B and 2C show the % ^99m^Tc and ^64^Cu positive uptake regions within tumors obtained from the analysis. The bevacizumab-treated tumors showed a significant decrease in % ^99m^Tc positive uptake regions (0.43-fold) and a significant increase in % ^64^Cu positive uptake regions within tumors (1.5-fold), compared to the bevacizumab-untreated control (*P* < 0.05). This indicates reduced vascularity and increased ^64^Cu-ATSM uptake regions within the bevacizumab-treated tumors.

### *In vivo* treatment study

**Figure 3:**
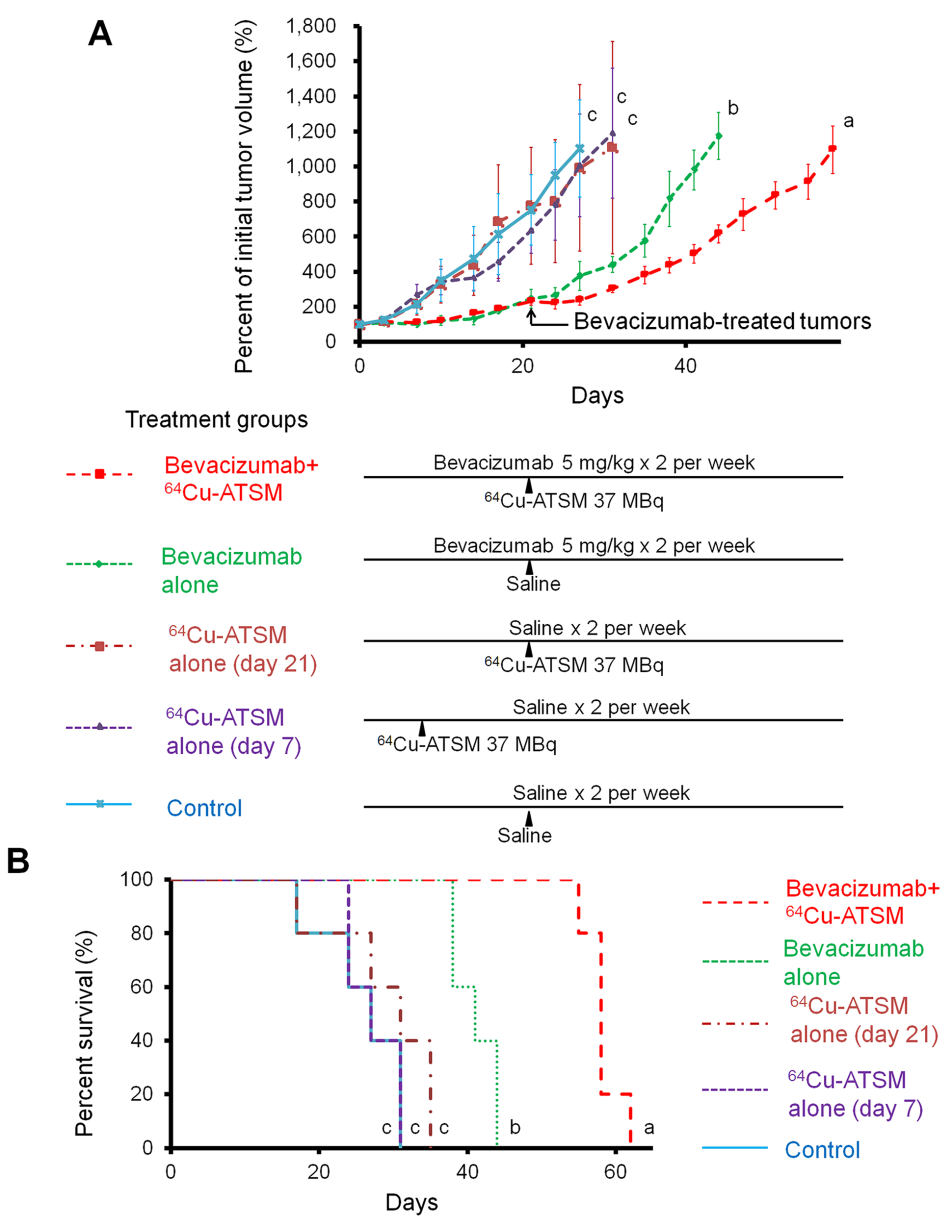
*In vivo* treatment study with ^64^Cu-ATSM The following groups were examined: bevacizumab+^64^Cu-ATSM; bevacizumab alone; ^64^Cu-ATSM alone-day 21; ^64^Cu-ATSM alone-day 7; control (n=5/group). **(A)** Tumor growth curves. Treatment schedule is also shown. Values are shown as mean ± SD. a, b, c; Different letters indicate significant differences (*P* < 0.05). **(B)** Survival curves. a, b, c; Different letters indicate significant differences (*P* < 0.05).

**Figure 4:**
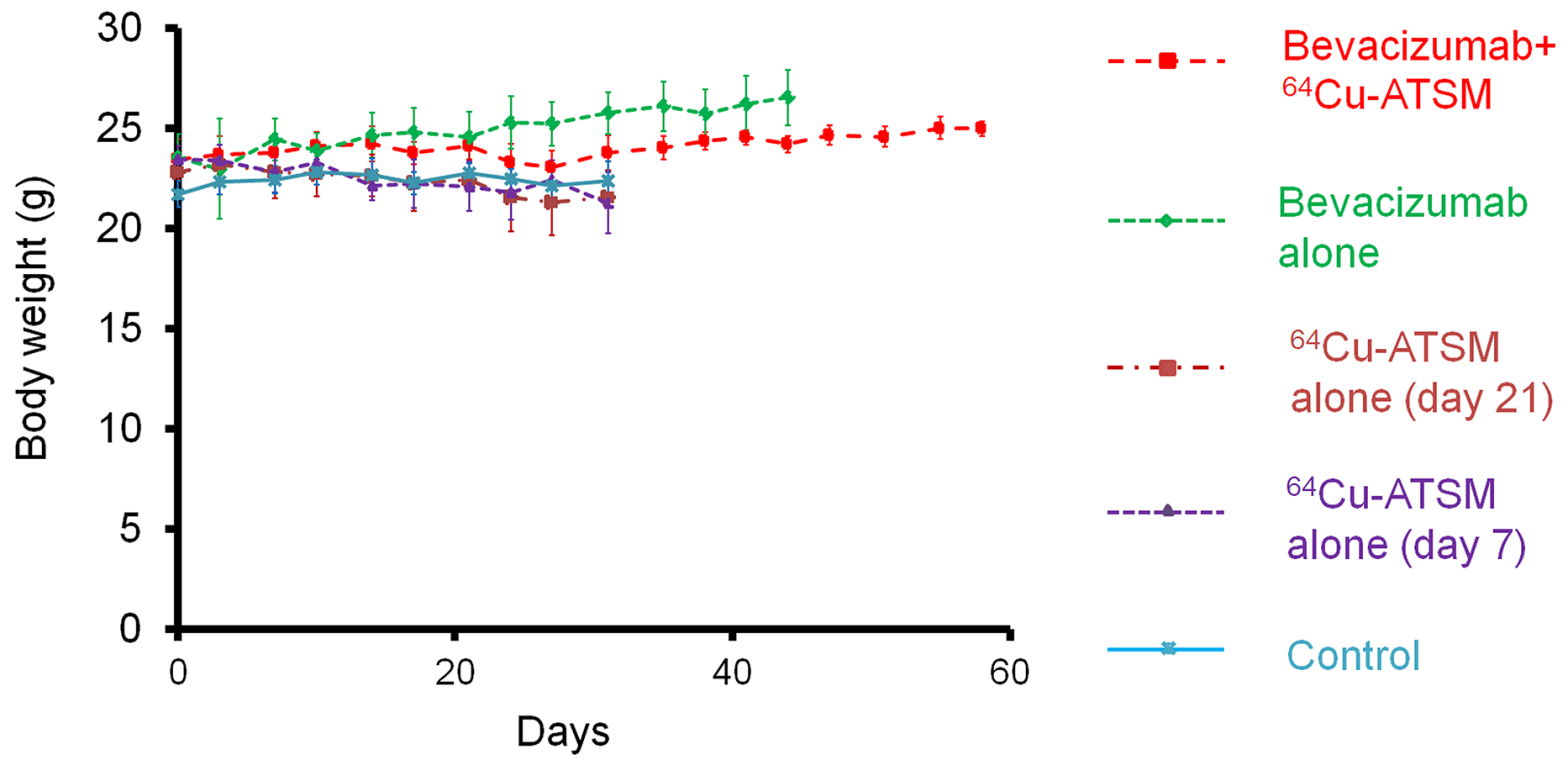
Body weight change in *in vivo* treatment study Body weight measured during *in vivo* treatment study shown in Figure 3. There was no weight loss greater than 10% of the initial body weight in any treatment group.

**Figure 5:**
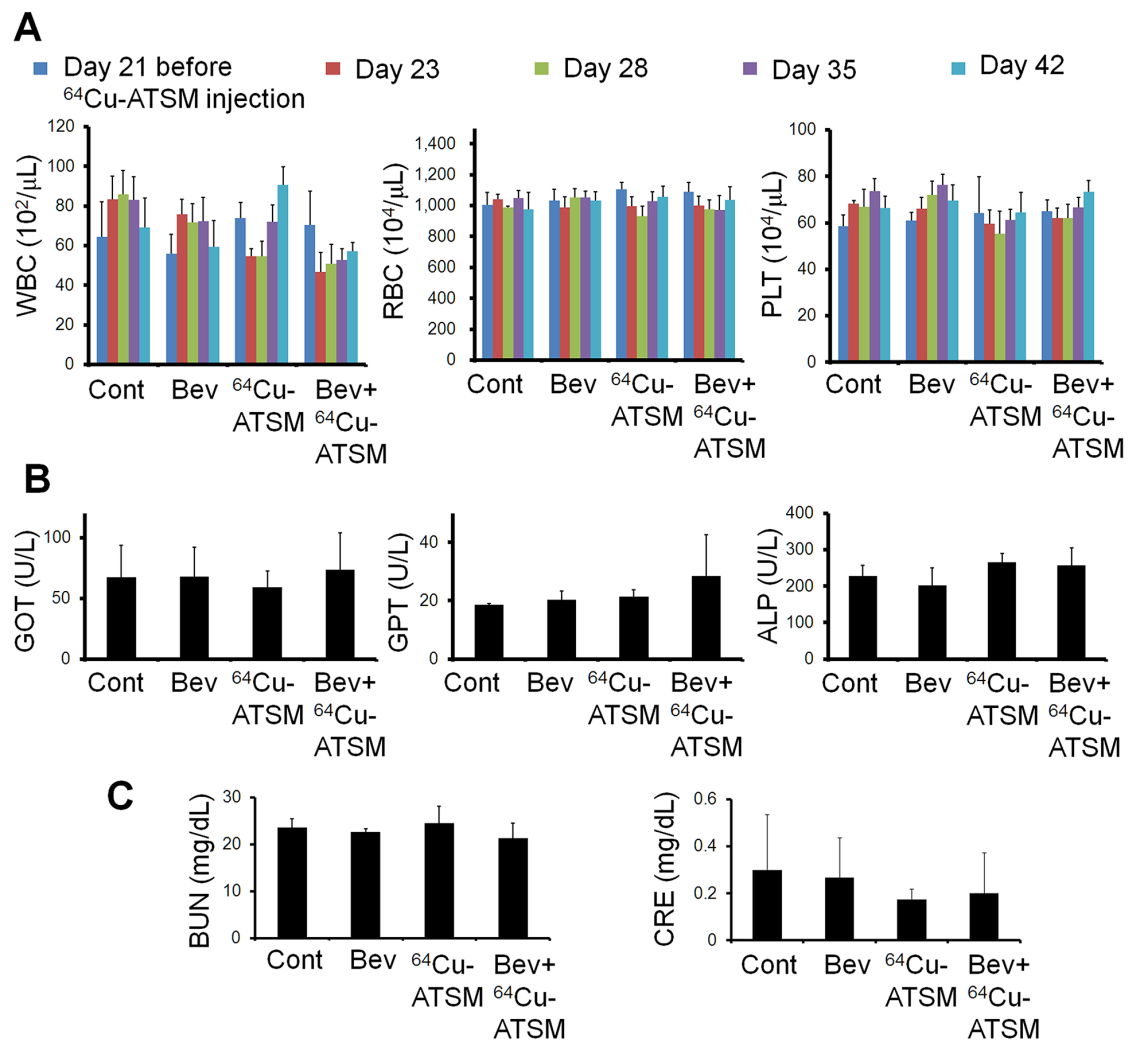
Hematological and hepatorenal toxicity **(A)** Number of white blood cells (WBC), red blood cells (RBC), and platelets (PLT) at the starting point before ^64^Cu-ATSM injection and at 2, 7, 14, and 21 days after ^64^Cu-ATSM injection (day 21 and days 23, 28, 35, and 42 in *in vivo* treatment study, respectively). **(B)** and **(C)** Levels of glutamate oxaloacetate transaminase (GOT), glutamate pyruvate transaminase (GPT), and alkaline phosphatase (ALP) for liver functions and urea nitrogen (BUN) and creatinine (CRE) for kidney function measured at 21 days after ^64^Cu-ATSM injection (day 42 in treatment study). Bev, bevacizumab.

Next, the efficacy of ^64^Cu-ATSM treatment against the bevacizumab-treated tumors was examined *in vivo*. For treatment study, HT-29 tumor-bearing mice were treated with bevacizumab (5 mg/kg twice a week) for three weeks to make the bevacizumab-treated tumor model as described above. The mice were intravenously injected with ^64^Cu-ATSM (37 MBq) or saline (day 21) and continued to be treated with bevacizumab (bevacizumab+^64^Cu-ATSM group or bevacizumab alone). The dose of ^64^Cu-ATSM (37 MBq) used in this study was decided based on toxicity and dosimetry analyses of previous studies, which demonstrated that the dosage results in no significant body weight loss and suggested not to administer radiation doses on the dose-limiting organs beyond the tolerance level [16, 26, 30]. For comparison, groups without bevacizumab treatment were also examined: ^64^Cu-ATSM (37 MBq) on day 21 (^64^Cu-ATSM alone-day 21 group); ^64^Cu-ATSM (37 MBq) on day 7, on which day tumor size was similar to that in the bevacizumab-treated mice on day 21 (^64^Cu-ATSM alone-day 7 group); saline instead of ^64^Cu-ATSM (day 21) (control group). In the groups without bevacizumab, saline was injected instead of bevacizumab. Figure 3A shows the change in relative tumor volume with time. Treatment with bevacizumab+^64^Cu-ATSM showed the greatest inhibition of tumor growth in the examined groups compared to the other groups (*P* < 0.05). Bevacizumab alone also showed significantly greater inhibition compared to ^64^Cu-ATSM alone on days 7 and 21 as well as the control group. ^64^Cu-ATSM alone appeared to inhibiting tumor growth to a higher extent than the control, although no significant difference was observed. Tumor growth time and tumor growth delay for each group are shown in Supplementary Table 1. Bevacizumab+^64^Cu-ATSM prolonged tumor growth time by 25.2 days compared to the control group. In contrast, bevacizumab alone and ^64^Cu-ATSM alone (day 21) prolonged tumor growth time by 15.1 days and 2.8 days compared to the control group, respectively (Supplementary Table 1). This means that tumor growth delay of bevacizumab+^64^Cu-ATSM was much longer than the sum of the tumor growth delay of bevacizumab alone and ^64^Cu-ATSM alone (day 21), which indicates that co-administration of bevacizumab and ^64^Cu-ATSM synergistically inhibited tumor growth compared to each single treatment. Figure 3B shows the survival curves. Overall survival following bevacizumab+^64^Cu-ATSM treatment (58.2 ± 2.2 days) was significantly greater than that observed for bevacizumab alone (41.0 ± 2.7 days), ^64^Cu-ATSM alone-day 21 (29.0 ± 6.7 days), ^64^Cu-ATSM alone-day 7 (27.4 ± 3.1 days), and the control (26.0 ± 5.2 days) (*P* < 0.05). A synergistic effect on overall survival was also shown in the co-administration of bevacizumab and ^64^Cu-ATSM, compared to each single treatment. Weight loss greater than 10% of the initial body weight was not observed in any treatment group (Figure 4). To evaluate side effects, hematological and biochemical parameters were also measured using tumor-free mice that received similar treatments to the groups of control, bevacizumab alone, ^64^Cu-ATSM alone-day 21, and bevacizumab+^64^Cu-ATSM in the *in vivo* treatment study, respectively. There were no significant reductions in the number of white blood cells, red blood cells, and platelets at any examined time point in the all treatment groups, compared to the starting point (day 21) in the control (Figure 5A). There were no significant differences in any parameters of glutamate oxaloacetate transaminase, glutamate pyruvate transaminase, and alkaline phosphatase for liver function, and urea nitrogen and creatinine for kidney function, when compared to the control (Figure 5B and 5C). These results indicate co-administration of bevacizumab and ^64^Cu-ATSM did not cause adverse effects in mice.

## DISCUSSION

We reported here that ^64^Cu-ATSM effectively inhibited the growth of HT-29 tumors with bevacizumab-induced vascular decrease and hypoxia, and prolonged the survival of the mice during bevacizumab treatment with negligible toxic side effects. In recent years, bevacizumab has been widely used in antiangiogenesis therapy in clinical practice. Previous studies showed that bevacizumab treatment is known to prolong patient survival; however, repeated use of bevacizumab leads to the therapeutic ineffectiveness [3]. Bevacizumab initially inhibits tumor growth, but continuous use of this agent makes it less effective due to reduced drug delivery and induced hypoxia with activation of an HIF-1 signaling pathway [3–5]. In the present study, we demonstrated that ^64^Cu-ATSM therapy can be a beneficial approach to treat the tumors with bevacizumab-induced vascular decrease and hypoxia.

The bevacizumab-treated tumors used for this study showed decreased blood vessel density and activation of HIF-1 signaling pathway. Despite the low blood vessel density, uptake of ^64^Cu-ATSM increased in the tumors. The imaging results showed that ^99m^Tc positive areas were decreased and ^64^Cu positive areas were increased in bevacizumab-treated tumors. These data indicate that bevacizumab treatment reduced vascularity and activated hypoxia-induced HIF-1 signaling in the tumors, and that ^64^Cu-ATSM can be delivered into such tumors in which drug delivery is probably limited. ^64^Cu-ATSM has a high tissue permeability and can be accumulated in hypoxic tissues even where blood perfusion is limited [9, 17]. This high permeability is likely to overcome the difficulties encountered during bevacizumab therapy.

Our results demonstrated that ^64^Cu-ATSM can inhibit tumor growth in bevacizumab-treated tumors. Our data also indicate that ^64^Cu-ATSM has synergistic effect on bevacizumab treatment. The increased tumor uptake of ^64^Cu-ATSM and expansion of ^64^Cu positive areas within the bevacizumab-treated tumors could be one reason for the synergistic therapeutic effect of ^64^Cu-ATSM on bevacizumab treatment. In addition, bevacizumab is known to have radiosensitizing effects by inhibiting both the VEGR2/PI3K/Akt/DNA-PKcs signaling pathway and DNA double strand break repair after irradiation [31]. The radiosensitizing effect of bevacizumab on ^64^Cu-ATSM might also contribute to the synergistic inhibitory effect of ^64^Cu-ATSM on the growth of the tumors.

Our *in vivo* treatment study showed that administration of 37 MBq of ^64^Cu-ATSM had a therapeutic effect against bevacizumab-treated tumors without noticeably increased toxicity levels. We have previously demonstrated that 37 MBq of ^64^Cu-ATSM was an appropriate dosage for treatment in mice, since it did not cause significant body weight loss and hematotoxicity [16, 26, 30]. In addition, we have estimated the human dosimetry of ^64^Cu-ATSM based on the time-series biodistribution data in normal organs and tumors using mice bearing HT-29 tumors [16, 30]. The dosimetry studies have demonstrated that liver, red marrow and ovaries were the dose-limiting organs in ^64^Cu-ATSM therapy and that the estimated radiation doses to those organs in human at the therapeutic dosage of ^64^Cu-ATSM calculated from 37 MBq per mouse were low enough compared to the reported tolerance doses [16, 30]. These findings suggest that the ^64^Cu-ATSM therapy during the bevacizumab treatment could be applied to humans. However, careful determination of the administration dosage and timing for ^64^Cu-ATSM is necessary for human study.

This study has several limitations. Optimal administration timing as well as multiple dosages of ^64^Cu-ATSM were not examined. These issues should be considered in future preclinical and clinical studies to achieve a more efficient and effective outcome of this combination therapy. In addition, we used a human colon carcinoma HT-29 tumor-bearing mouse model, because colon cancer is one of the most popular target for bevacizumab [1]. The efficacy of the ^64^Cu-ATSM with bevacizumab in the other types of cancer would be warranted by further studies.

In conclusion, this study demonstrates that ^64^Cu-ATSM effectively inhibited tumor growth and prolonged the survival without major toxicity in mice bearing tumors with bevacizumab-induced vascular decrease and hypoxia. These findings suggest that ^64^Cu-ATSM therapy has potential as a new therapeutic option for tumors with long-term bevacizumab treatment in clinical practice.

## MATERIALS AND METHODS

### Preparation of ^64^Cu-ATSM

^64^Cu was produced, purified, and used for synthesis of ^64^Cu-ATSM according to previously reported procedures [16, 32]. The radiochemical purity of ^64^Cu-ATSM was determined by silica gel thin-layer chromatography (TLC; silica gel 60; Merck) with ethyl acetate as the mobile phase [33]. Radioactivity on the TLC plates was analyzed with a bioimaging analyzer (FLA-7000; Fujifilm). The radiochemical purity of ^64^Cu-ATSM was found to be greater than 95%.

### Cell culture and animal model

Human colon carcinoma HT-29 cells (HTB-38; American Type Culture Collection) were cultured under standard conditions in Dulbecco’s modified Eagle’s medium (Invitrogen) supplemented with 10% fetal bovine serum.

All animal experimental procedures were approved by the Animal Ethics Committee of the National Institutes for Quantum and Radiological Science and Technology (QST, Chiba, Japan) and conducted in accordance with the institutional guideline. Six-week-old male BALB/c nude mice (20–25 g body weight) were obtained from Japan SLC. HT-29 cells (1 × 10^7^ cells) suspended in phosphate-buffered saline (PBS) were subcutaneously injected into the flanks of the mice. Mice bearing tumors approximately 5 mm in diameter were used for subsequent experiments. To make a bevacizumab-treated tumor model with vascular decrease and hypoxia, HT-29 tumor-bearing mice were treated with bevacizumab (Chugai) (5 mg/kg twice a week) for 3 weeks. To assess decreased vascularity and hypoxia within the tumors treated with bevacizumab for 3 weeks, characterization and imaging studies were performed prior to the treatment study, as follows. Untreated tumors, which reached similar size to the bevacizumab-treated tumors on day 21, were used for control (bevacizumab-untreated control) in the characterization and imaging studies.

### Characterization study

To examine blood vessel density, immuno-histochemistry for CD31, a blood vessels marker, was performed using standard protocol with the bevacizumab-treated HT-29 tumors and bevacizumab-untreated control (*n* = 4). Details are described in Supplementary Methods. Images were obtained with a Leica DM 2500 microscope (Leica). Four observation areas (1155 μm×1530 μm) were randomly selected from each tumor section of four different tumors from each group and the number of blood vessels in the observation areas was determined.

To examine the hypoxia-induced reaction, we evaluated activation of the HIF-1 signaling pathway by DNA microarray-based analysis as reported previously [16], with bevacizumab-treated HT-29 tumors, and compared to the bevacizumab-untreated controls (n=3/group). Details of DNA microarray and pathway analysis are described in Supplementary Methods. The DNA microarray data were deposited in the Gene Expression Omnibus database under accession number GSE86525.

^64^Cu-ATSM uptake in tumors was examined and compared between mice with the bevacizumab-treated HT-29 tumors and the bevacizumab-untreated control group. Mice were intravenously injected with a tracer dose of ^64^Cu-ATSM (185 kBq/mouse) (n=4/group) and sacrificed 1 h after the injection. The tumors were collected and weighed. Radioactivities were counted with a γ-counter (1480 Automatic gamma counter Wizard 3; PerkinElmer). The results were shown as %ID/g.

### *In vivo* high-resolution simultaneous SPECT/PET/CT imaging

To examine the intratumoral vascularity and ^64^Cu-ATSM uptake regions, dual-isotope simultaneous SPECT/PET/CT imaging with ^99m^Tc-HSA and ^64^Cu-ATSM was performed using a method previously reported [29], with bevacizumab-treated HT-29 tumors and the bevacizumab-untreated control (n=4/group). ^99m^Tc-HSA was prepared using ^99m^Tc-pertechnetate (Nihon Medi-Physics) and Techne Albumin Kit (Fujifilm RI Pharma) according to the manufacturer’s protocol [29]. The VECTor small-animal scanner (MILabs), with a tungsten collimator and NaI(Tl) crystal detectors, which allows *in vivo* high-resolution SPECT/PET/CT imaging [29], was used in this study. For the imaging study, HT-29 cells suspended in PBS (1 × 10^7^ cells) were subcutaneously injected into the femoral region of the right hind leg to fit the image acquisition using the stage of this system. Fifty min after the intravenous injection of ^64^Cu-ATSM (37 MBq), ^99m^Tc-HSA (18.5 MBq) was intravenously injected into the mouse. 10 min after ^99m^Tc-HSA administration, simultaneous SPECT/PET/CT scanning was performed for 60 min, focusing on the tumor regions. Injection dose and timing of these tracers were decided based on previous studies [30, 34, 35]. Each probe was dissolved in 100 μl of saline. During the scans, the mice remained under 2% isoflurane anesthesia, and body temperature was maintained with a heater. The mouse PET 0.7 collimator, which contains 48 clusters of four 0.7-mm diameter pinholes placed in 4 rings, and whose central field of view is approximately 12 mm in diameter and 9 mm in longitudinal length [36], was used in this study. Simultaneous SPECT/PET/CT scan was performed by list-mode acquisitions using the manufacturer’s software (version 3.6g3s, MILabs). For CT, non-contrast-enhanced acquisitions were performed with the following parameters: 60 kV tube voltage, 615 μA tube current, partial scan angle, and fast scan mode. The SPECT/PET projections were reconstructed with a pixel-based, ordered-subset expectation maximization algorithm [37] using VECTor software (version 2.38c). The triple-energy window method [38] was used for scatter correction with the following parameters: for ^99m^Tc, photo peak window was set to a width of 29%; background = 10% on left side and 7% on right side of the photo peak; 16 subsets; 15 iterations; 0.4-mm voxel size; no filter; for ^64^Cu, photo peak window was set to a width of 30.2%; background = 10% on left side and 7% on right side of the photo peak; 32 subsets; 15 iterations; 0.4-mm voxel size; and no filter. These parameters were decided based on [36, 39]. Registration of the reconstructed SPECT, PET, and CT images was performed with the software (version 2.38c). Registered SPECT/PET/CT images were analyzed by the biomedical image quantification software PMOD (PMOD Technologies). Radioactivity density values (kBq/cc) on SPECT and PET images were determined based on calibration with a known activity concentration using the decay correction. With the reconstructed and registered images, the volumes of interest (VOIs) were positioned to cover the tumor regions. The standardized uptake values (SUVs), the mean activity concentration divided by the injected activity per body weight, were calculated for each pixel in the VOIs. The regions with SUVs of 0.5 or more were regarded as positive uptake regions and the percentage of ^99m^Tc and ^64^Cu positive uptake regions within the VOIs was calculated, respectively.

### *In vivo* treatment study

Mice bearing HT-29 tumors were randomized into five groups (n=5/group). Tumor-bearing mice were treated with bevacizumab (5 mg/kg twice a week) for three weeks to make the bevacizumab-treated tumor model as described above. They were intravenously injected with ^64^Cu-ATSM (37 MBq) or saline (day 21) and continued to be treated with bevacizumab (bevacizumab+^64^Cu-ATSM group or bevacizumab alone). Also, the following three groups without bevacizumab treatment were examined for comparison: ^64^Cu-ATSM (37 MBq) on day 21 (^64^Cu-ATSM alone-day 21 group); ^64^Cu-ATSM (37 MBq) on day 7, on which day tumor size was similar to that in the bevacizumab-treated mice on day 21 (^64^Cu-ATSM alone-day 7 group); saline instead of ^64^Cu-ATSM (day 21) (control group). In the groups without bevacizumab, saline was injected instead of bevacizumab. During the *in vivo* treatment study, mice were weighed, and tumor size was measured using precision calipers twice weekly. Tumor volume was calculated using an equation, tumor volume = length × width^2^ × π/6, and the relative tumor volume was calculated by dividing the tumor volume on each day by that on day 0. The initial tumor volume is shown in Supplementary Table 2. Mice were sacrificed when the tumor volume reached the humane endpoint. Based on the growth curve, tumor growth time and tumor growth delay were also analyzed. Tumor growth time was defined as time in days necessary to reach a 5-fold increase of individual tumor volume from the initial volume. Tumor growth delay was calculated as the difference in mean tumor growth time between the treatment groups and the control.

### Measurement of hematological and biochemical parameters

For evaluation of side effects, hematological and biochemical parameters were examined using tumor-free mice that received similar treatments to the groups of control, bevacizumab alone, ^64^Cu-ATSM alone-day 21, and bevacizumab+^64^Cu-ATSM in the *in vivo* treatment study, respectively (n=4/group). Measurements of hematological parameters were performed at the starting point just before ^64^Cu-ATSM injection (day 21 in the treatment study) and 2, 7, 14, and 21 days after ^64^Cu-ATSM injection (day 23, 28, 35, and 42 in the treatment study), using blood collected from a tail vein. Concentration of white blood cells, red blood cells, and platelets was determined using a hematological analyzer (Celltac MEK-6458, Nihon Kohden). Biochemical parameters were measured 21 days after ^64^Cu-ATSM injection (day 42 in the treatment study), using mouse plasma prepared with blood collected from the heart. Levels of glutamate oxaloacetate transaminase, glutamate pyruvate transaminase, and alkaline phosphatase for liver function as well as urea nitrogen and creatinine for kidney function were measured using a blood biochemical analyzer (Dri-Chem 7000VZ, Fuji Film).

### Statistical analysis

Data were expressed as means with corresponding standard deviations. *P* values were calculated using a two-sided *t*-test for comparisons between two groups. Analysis of variance (ANOVA) was used for comparisons between multiple groups. Tumor growth curves were analyzed using a two-way ANOVA. Differences in survival were evaluated by the log-rank test. *P* values less than 0.05 were considered statistically significant.

## SUPPLEMENTARY MATERIALS TABLES


