# Supplementary material for: 64Cu-ATSM internal radiotherapy to treat tumors with bevacizumab-induced vascular decrease and hypoxia in human colon carcinoma xenografts
Source: Oncotarget. 2017 Sep 28;8(51):88815–26. doi: 10.18632/oncotarget.21323 (PMC5687648; doi:10.18632/oncotarget.21323)
Supplement: Supplementary file 1 [file oncotarget-08-88815-s001.pdf]

^{64}Cu-ATSM internal radiotherapy to treat tumors with bevacizumab-induced vascular decrease and hypoxia in human colon carcinoma xenografts

SUPPLEMENTARY MATERIALS

SUPPLEMENTARY METHODS

Immunohistochemistry for blood vessel density

To examine blood vessel density, immunohistochemistry for CD31 was performed with the bevacizumab-treated HT-29 tumors and the untreated control (n = 4). The isolated tumors were fixed with 10% buffered formalin for 2 days at room temperature, processed for paraffin embedding, and sectioned according to standard histological procedures. Sections (6 µm) were cut. After deparaffinization and dehydration, the sections were microwaved for antigen retrieval and placed in a solution of 0.3% hydrogen peroxide in methanol for 30 min to quench endogenous peroxidase. To block nonspecific binding, a protein-blocking agent (G-Block, Genostaff) was applied for 10 min at room temperature. The endogenous avidin and biotin activities were blocked using the Avidin/Biotin Blocking Kit (Vector). The sections were incubated overnight at 4 °C with a primary antibody against CD31 (1:50 dilution; E11110, Spring Bioscience). After washing the sections with Tris-buffered saline (TBS), biotinylated secondary antibody (E0432, Dako) was applied for 30 min at room temperature. After another TBS wash, the sections were incubated with streptavidin peroxidase reagent (426062, Nichirei) for 5 min and washed with TBS. Color development was performed using 3,3'-diaminobenzine tetrahydrochloride solution with H_2O_2 after which the sections were counterstained with hematoxylin.

DNA microarray-based analysis

Total RNA was extracted from samples with the PureLink RNA Mini Kit (Life Technologies). The integrity of the RNA was evaluated using an Agilent 2100 Bioanalyzer (Agilent Technologies). The Low Input Quick Amp Labeling Kit, one-color (Agilent Technologies) was used to prepare Cy3-labeled target cRNA according to the manufacturer's instructions. Labeled cRNAs were hybridized with a SurePrint G3 Human GE 8×60K Microarray (Agilent Technologies). Array images were captured using a DNA Microarray Scanner (Agilent Technologies), and the data was analyzed using Feature Extraction Software (Agilent Technologies) to obtain background-corrected signal intensities. Date was further analyzed with GeneSpring GX Software (Version 11.0; Agilent Technologies). After data filtering, mRNAs differentially expressed in the bevacizumab-treated HT-29 tumors when compared to the bevacizumab-untreated control were assessed by Fisher's exact test, followed by multiple corrections using the Benjamini and Hochberg false discovery rate method. Gene sets with a q-value less than 0.05 were considered to be significant. For the pathway analysis, we used the functions for finding significant pathways in GeneSpring GX Software package. The DNA microarray data was deposited in the Gene Expression Omnibus database under accession number GSE86525.

Supplementary Table 1: Analysis on tumor growth time and tumor growth delay in *in vivo* treatment study with HT-29 tumors

Treatment	Mean tumor growth time[1] (day)	Tumor growth delay[2] (days)
Bevacizumab+^{64}Cu-ATSM	42.2 ± 2.4	25.2
Bevacizumab alone	32.1 ± 1.8	15.1
^{64}Cu-ATSM alone-day 21	19.8 ± 6.0	2.8
^{64}Cu-ATSM alone-day 7	20.7 ± 2.9	3.7
Control	17.0 ± 4.4	-

[1]Tumor growth time was determined as time in days necessary to gain a fivefold increase in individual tumor volume from the size at the start of treatment, which was calculated based on the growth curve of each tumor.
[2]Tumor growth delay was calculated as differences in mean tumor growth time between treatment groups vs control.

Supplementary Table 2: Initial tumor volume in the *in vivo* treatment study

Treatment	Initial tumor volume (mm^3)[1]
Bevacizumab+^{64}Cu-ATSM	73.35 ± 8.00
Bevacizumab alone	80.38 ± 18.01
^{64}Cu-ATSM alone-day 21	79.11 ± 14.80
^{64}Cu-ATSM alone-day 7	83.52 ± 9.93
Control	79.11 ± 14.80

[1]There were no significant differences among groups.
